# Association between the point-rating system used for oral health and the prevalence of Gramnegative bacilli in hematological inpatients: A retrospective cohort study: Erratum

**DOI:** 10.1097/MD.0000000000026493

**Published:** 2021-06-25

In the article, “Association between the point-rating system used for oral health and the prevalence of Gramnegative bacilli in hematological inpatients: A retrospective cohort study”,^[1]^ which appears in Volume 100, Issue 22 of *Medicine*, the author list appears incorrectly. It has been updated from “Kunio Yoshizawa, DDS, PhDa,∗, Akinori Moroi, DDS, PhDa, Ran Iguchi, DDS, PhDa, Hiroshi Yokomichi, MD, PhDb, Shinji Ogihara, MT, PhDc, Kei Nakajima, MD, PhDd, Keita Kirito, MD, PhDd, Koichiro Ueki, DDS, PhDa” to “Kunio Yoshizawa, DDS, PhDa,∗, Akinori Moroi, DDS, PhDa, Ran Iguchi, DDS, PhDa, Hiroshi Yokomichi, MD, PhDb, Shinji Ogihara, MT, PhDc, **Kazuaki Watanabe, MT**^**c**^, Kei Nakajima, MD, PhDd, Keita Kirito, MD, PhDd, Koichiro Ueki, DDS, PhDa.”
